# Down-Turner Syndrome: A Case with Double Monoclonal Chromosomal Abnormality

**DOI:** 10.1155/2016/8760504

**Published:** 2016-09-08

**Authors:** Gioconda Manassero-Morales, Denisse Alvarez-Manassero, Alfredo Merino-Luna

**Affiliations:** ^1^Genetics Division, Instituto Nacional de Salud del Niño, San Borja, Peru; ^2^School of Medicine, Universidad Peruana de Ciencias Aplicadas, Lima, Peru

## Abstract

*Introduction*. The coexistence of Down and Turner syndromes due to double chromosome aneuploidy is very rare; it is even more rare to find the presence of a double monoclonal chromosomal abnormality.* Objective*. To report a unique case of double monoclonal chromosomal abnormality with trisomy of chromosome 21 and an X ring chromosome in all cells studied; no previous report has been found.* Case Report*. Female, 28 months old, with pathological short stature from birth, with the following dysmorphic features: tilted upward palpebral fissures, short neck, brachycephaly, and low-set ears. During the neonatal period, the infant presented generalized hypotonia and lymphedema of hands and feet. Karyotype showed 47,X,r(X),+21 [30].* Conclusion*. Clinical features of both Down and Turner syndromes were found, highlighting short stature that has remained below 3 *z* score from birth to the present, associated with delayed psychomotor development. G-banded karyotype analysis in peripheral blood is essential for a definitive diagnosis.

## 1. Background

The incidence of Down syndrome is 1 in 700 newborns, while the incidence of Turner syndrome is 1 in 5,000 births. The coexistence of double aneuploidy is very rare; Down-Turner published reports showed mosaicism related to two or more cell lines; the first case was reported in 1971 [1–3].

Subsequently, other reports of cases of trisomy 21 combined with Turner syndrome showed different cytogenetic variants [4–9]; the most frequent was mosaicism of two clonal lines, one clone with trisomy 21 and another with X monosomy, with an incidence of 1 in 2,000,000 births [5]. Associations with hemangioma [6] or congenital knee dislocation [7] have been described. Down-Turner syndrome has never been reported in Latin America and we did not found any report of double monoclonal chromosomal abnormality with trisomy 21 plus structural abnormality of the X chromosome.

## 2. Clinical Case

A term female newborn, product of a third gestation, was delivered at 39 weeks via cesarean section due to rupture of membranes without labor. Weight was 2.800 kilograms and height was 44 centimeters at birth. She presented lymphedema of hands and feet and jaundice requiring phototherapy from second to fourth day of life.

In the first pediatric control, at one month, generalized hypotonia was detected, and physical therapy was recommended. Delayed psychomotor development was noted, achieving a sitting position at 10 months and standing at 24 months. The baby was referred to the genetics clinic because of delay psychomotor development and short stature.

At the age of 2 years and 4 months, anthropometric data showed size of 77 cms (−3.31 *z* score), weight of 10.15 kilos (−2.26 *z* score), and head circumference of 43 cms (−3.25 *z* score); some dysmorphic features as tilt upward palpebral fissures, short neck, brachycephaly, and low-set ears were reported.

**Figure 1:**
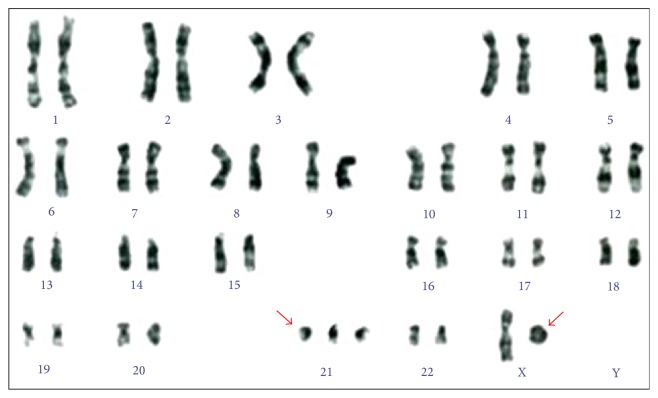
47,X,r(X),+21 [30].

Peripheral blood karyotype by cytogenetic banding G analysis showed 47,X,r(X),+21 [30] (Figure 1).

## 3. Discussion

We report this case because no report of a double monoclonal chromosomal abnormality has been found with coexistence of autosomal trisomy and structural abnormality of an X chromosome. Furthermore, Down-Turner syndrome has never been reported in Latin America. Clinical diagnosis of Down syndrome is at birth, according to the characteristic phenotypic traits. Short stature at birth and edema of hands and feet are clinical features that suggest Turner syndrome. However, not always the dysmorphic features are evident in the neonatal stage, and sometimes they are unnoticed on clinical examination. The diagnosis of autosomal and/or sex chromosomes aneuploidies or other structural abnormalities can be made postnatal [1–9] or prenatal [10, 11].

## 4. Conclusion

Pathological short stature from birth, delayed psychomotor development, and the presence of certain dysmorphic features should alert the pediatrician to request a cytogenetic study. However, it is necessary to mention that this diagnostic tool is not available in all hospitals in our country.
